# Leveraging Medical Claims to Predict Long-term Care Transitions among Older Adults in the United States

**DOI:** 10.1093/geroni/igab046.3411

**Published:** 2021-12-17

**Authors:** Megan Backhaus, An-Ting Jhuang, Ben Griffith, Lauren Bangerter

**Affiliations:** 1 UnitedHealth Group, Minnetonka, Minnesota, United States; 2 OptumLabs, Eden Prairie, Minnesota, United States

## Abstract

Most older adults prefer to age in place rather than moving to a long-term care (LTC) facility, but little is known about the factors that predict entry into LTC. This study sought to utilize administrative claims data to understand the predictors of LTC transitions using de-identified claims data from Medicare Advantage members in the UnitedHealth Group Clinical Discovery Database. We investigated LTC transitions of 250,587 adults (Mean age = 77, standard deviation = 7.75) between January 1, 2016 and December 31, 2019. Types of predictors for these transitions include aggregated medical data surrounding chronic conditions and frailty indices, as well as healthcare utilization and demographics in 2016 and 2017. We then fit data of these types to an extreme gradient boosting (XGBoost) model to predict long-term care transitions in 2018 and 2019 (ROCAUC = 0.84, accuracy = 0.84, precision = 0.68, and recall = 0.42). Frailty indicators, such as falls and fractures, mobility problems, dementia, and delirium, as well as osteoporosis are strong predictors of LTC transitions. These findings can be used to design interventions aimed at preventing LTC transitions and enabling older adults to age in place.

